# Linguistically annotated dataset for four official South African languages with a conjunctive orthography: IsiNdebele, isiXhosa, isiZulu, and Siswati

**DOI:** 10.1016/j.dib.2022.107994

**Published:** 2022-02-25

**Authors:** Tanja Gaustad, Martin J. Puttkammer

**Affiliations:** Centre for Text Technology, North-West University, South Africa

**Keywords:** Natural language processing, Human language technology, Linguistic annotation, Nguni languages

## Abstract

This data article presents a linguistically annotated data set for four official South African languages with a conjunctive orthography, namely isiNdebele, isiXhosa, isiZulu and Siswati. The data set is parallel for all four languages and can be used for language-specific as well as cross-language development and evaluation of Natural Language Processing (NLP) core technologies. In addition, it can be used for corpus linguistic studies. The article describes how the data was collected, what type of texts it contains and it provides some details on the three different types of linguistic annotation added (morphology, part-of-speech and lemmas), including an example.

## Specifications Table

**Table:** 

Subject	Computer Science
Specific subject area	Natural Language Processing, Human Language Technology, Linguistic corpus
Type of data	Textual data with 3 different types of linguistic annotations (linguistically annotated corpus) for 4 Nguni languages
How data were acquired	The data is a cleaned-up subset of a collection of documents originating from the South African government domain websites (*.gov.za) that contains parallel data for isiZulu, isiXhosa, isiNdebele and Siswati.
Data format	Raw (ASCII) text, analysed (annotated), parallel
Parameters for data collection	All data included in the dataset had to be present in all four languages. Also, data containing only contact information, repetition of whole sentences, lists and headings was removed.
Description of data collection	The South African government domain websites (*.gov.za) were crawled. The retrieved documents in pdf and Microsoft Word format were converted to text format, language identification was performed and the files were aligned on paragraph-level across the relevant languages. After clean-up, the content was split into paragraphs, sentences and tokens before the linguistic annotations were added.
Data source location	Institution: Centre for Text Technology, North-West University City/Town/Region: Potchefstroom Country: South Africa
Data accessibility	Repository name: SADiLaR Data identification number: NA Direct URL to data: https://repo.sadilar.org/handle/20.500.12185/546

## Value of the Data

• This parallel data set can be used for language-specific and cross-language development and evaluation of Natural Language Processing (NLP) core technologies and applications for isiZulu, isiXhosa, isiNdebele and Siswati.
• Researchers working on Human Language Technology (HLT) applications for the four Nguni languages (isiZulu, isiXhosa, isiNdebele and Siswati) will be able to use the dataset for research, development and evaluation purposes.
• The morphologically annotated data allow for better investigations of morphological phenomena that will in turn lead to new knowledge and insights into the processes involved in word creation in these languages.
• Part-of-speech annotated data is a common linguistic investigation attribute that is widely used in syntactic analysis, as well as more complex syntactic investigations.
• Lemmatisation is viewed as an indispensable linguistic information source and can also be utilised in various text processing applications such as spelling checkers, information retrieval systems, text mining and machine translation systems.
• Also, researchers in the fields of Linguistics and Digital Humanities will benefit from this parallel dataset for comparative corpus studies of Nguni languages.

## 1 Data Description

The dataset contains eight separate text files in UTF8 encoding, a train and a test set for four Nguni languages, namely isiZulu, isiXhosa, isiNdebele and Siswati. The unannotated English data is also provided as a reference. The start of each original paragraph is marked by a line marker with a counter. In total, the dataset contains 1431 paragraphs for each of the languages, all split randomly into a 90% training and 10% testing part. Every token-annotation combination can be found on a new line. Tokens and all three annotation types, specifically morphology, lemma and part-of-speech (POS), are separated by a single tab character resulting in a four-column data representation (see the example in Table 1). The annotations are described in separate protocols and form a finite set of tags, different for each annotation type. The morphological annotation contains alphabetic characters, numbers, punctuation marks, hyphens and square brackets. The lemma annotation contains alphabetic characters, punctuation marks and hyphens. The POS annotation contains uppercase characters and numbers.

**Table 1:** Example of annotated data in isiXhosa.

Token	Morphological analysis	Lemma	POS
<LINE# 0002>			
I-GEP	i[*NPrePre9*]-GEP[*Abbr*]	GEP	N09
inamancedo	i[*NPrePre9*]-na[*CopPre*]-ma[*BPre6*]-ncedo[*NStem*]	ncedo	N09
aliqela	a[*RelConc6*]-li[*BPre5*]-qela[*NStem*]	qela	REL
okuXhasa	a[*PossConc6*]-u[*NPrePre15*]-ku[*BPre15*]-xhas[*VRoot*]-a[*VerbTerm*]	xhasa	POSS06
ukuCwangcisa	u[*NPrePre15*]-ku[*BPre15*]-cwangcis[*VRoot*]-a[*VerbTerm*]	cwangcisa	V
awokukunceda	a[*RelConc6*]-wa[*PossConc6*]-u[*NPrePre15*]-ku[*BPre15*]-ku[*OC2ps*]-nced[*VRoot*]-a[*VerbTerm*]	nceda	REL
ukuqulunqa	u[*NPrePre15*]-ku[*BPre15*]-qulunq[*VRoot*]-a[*VerbTerm*]	qulunqa	V
izikhokelo	i[*NPrePre8*]-zi[*BPre8*]-khokelo[*NStem*]	khokelo	N08
zokulawula	za[*PossConc8*]-u[*NPrePre15*]-ku[*BPre15*]-lawul[*VRoot*]-a[*VerbTerm*]	lawula	POSS08
ishishini	i[*NPrePre5*]-(li)[*BPre5*]-shishini[*NStem*]	shishini	N05
lakho	la[*PossConc5*]-kho[*PossPron*]	kho	POSS05
nokuqaphela	na[*AdvPre*]-u[*NPrePre15*]-ku[*BPre15*]-qaphel[*VRoot*]-a[*VerbTerm*]	qaphela	ADV
izinto	i[*NPrePre10*]-zin[*BPre10*]-to[*NStem*]	to	N10
ezisilelayo	ezi[*RelConc10*]-silel[*VRoot*]-a[*VerbTerm*]-yo[*RelSuf*]	silela	REL
ezinokufuna	ezi[*RelConc10*]-na[*CopPre*]-u[*NPrePre15*]-ku[*BPre15*]-fun[*VRoot*]-a[*VerbTerm*]	funa	REL
kuthathwe	ku[*SC15*]-thath[*VRoot*]-w[*PassExt*]-e[*VerbTerm*]	thatha	V
inyathelo	i[*NPrePre5*]-(li)[*BPre5*]-nyathelo[*NStem*]	nyathelo	N05
.	.[*Punc*]	.	PUNC

The dataset and associated tag sets and protocols are distributed under the Creative Commons Attribution 4.0 International license.^1^

Table 2 contains the total token counts for each of the four languages.

**Table 2:** Statistics on annotations in the data.

Language	Token count	Unique tokens	Token hapax legomena	Unique lemmas	% POS tags used
isiNdebele	51,120	13,499	8677 (64.3%)	4241	91%
Siswati	48,816	14,142	8115 (57.4%)	2964	94%
isiXhosa	50,166	14,143	9157 (64.7%)	2254	96%
isiZulu	50,528	13,010	8297 (63.8%)	2669	92%
English	68,431	4926	1935 (39.3%)	NA	NA

## 2 Experimental Design, Materials and Methods

The initial dataset was created by randomly selecting documents from a collection of data originating from the South African government domain websites (*.gov.za) that contained parallel data for English, isiZulu, isiXhosa, isiNdebele and Siswati. The retrieved documents in pdf and Microsoft Word format were converted to text format using publicly available modules. Next, language identification was performed with a language identification tool for South African languages.^2^ The files were automatically aligned on paragraph level across the relevant languages and the alignments were manually verified. Clean-up was then performed to remove sections of the documents containing only contact information, repetition of whole sentences, lists and headings.

The text files consist of approximately 50,000 tokens total per language, parallel on paragraph-level between all four languages as well as English. English is used as the basis for the token counts during data selection due to the agglutinative nature of the four Nguni languages [1]. The final dataset contains 1431 paragraphs (for each of the languages) on different topics from the government domain, and includes speeches, press releases, health information and other information about government departments and services.

Each language corpus has then been annotated for three different types of linguistic information: morphology, part-of-speech (POS) and lemmas Table 1. contains an example for isiXhosa annotated data.

Before the annotation, we developed protocols and tag sets for all three levels of annotation which detail the procedure and standards used as well as the permissible tags. The tag sets and protocols are included in the data release. A total of 380 linguistically permissible morphology tags were defined, i.e. *[AdjPref10]* was used for the adjective prefix from class 10 or *[NStem]* to indicate a noun stem. These were combined during annotation to yield full morphological analyses of the tokens present in the data, e.g. *izinto* (‘things’) was analysed as *i[NPrePre10]-zin[BPre10]-to[NStem]*.

The POS tagset consists of 20 main word classes, e.g. *V* for verb or *N* for noun, with some classes including additional information on class numbers, e.g. *N08* (noun class 8) or *POSS10* (possessive class 10). A total of 107 unique POS tags was available to the annotators, most of which also occurred in the data (see Table 2).

For lemmatization, the aim was to identify the stem lemma for each token. In isiXhosa for example, the noun *izinto* will be analysed as the nominal stem *to*. This stem lemma in combination with the POS tag *N10* (Noun class 10) encodes the essential syntactic information for the word *izinto*.

Table 2 below summaries some statistics for the annotations contained in the data.

For each annotation task, the data was automatically pre-annotated and subsequently presented to linguistic experts for further annotation and correction in the CTexT Lara 2 annotation tool.^3^ The data was first annotated for morphology. Given the complex nature of agglutinative conjunctively written languages [2], the morphological information allowed for easier and more accurate pre-annotation of POS and lemma.

Once all levels of annotation had been completed, rigorous quality control (QC) was carried out to ensure that there is a high agreement between the morphology, POS and lemmatization annotations on token-level. The aim was to reach a 99% agreement on token-morph-lemma-POS combination. This was verified by using a rule-based generator to extrapolate the POS and lemma from the verified morphological analysis. The generated POS and lemma were then compared to the manually verified annotations received from the linguistic experts. Any discrepancies between the generated and QC'ed data was sent back to the linguistic experts to identify the error(s) and make corrections. Cross-language comparisons were also done to ensure the protocols were consistently applied across the four languages.

The final dataset was split into 90% train and 10% test sets. These datasets were used during the development of lemmatisers, POS taggers and morphological analysers for these languages, as described in [3] as well as for the South African Conference for Artificial Intelligence Research (SACAIR) and the Digital Humanities Association of Southern Africa (DHASA) shared task, the 2021 Nguni languages POS tagging challenge.^4^

## Ethics Statement

NA.

## Data Availability

Linguistically enriched corpora for conjunctively written South African languages (Original data).

## CRediT authorship contribution statement

**Tanja Gaustad:** Data curation, Methodology, Supervision, Validation, Writing – original draft, Writing – review & editing. **Martin J. Puttkammer:** Conceptualization, Funding acquisition, Methodology, Writing – review & editing.

## Declaration of Competing Interest

The authors declare that they have no known competing financial interests or personal relationships which have or could be perceived to have influenced the work reported in this article.

## Footnotes

1 CC BY 4.0 - https://creativecommons.org/licenses/by/4.0/.

2 https://hdl.handle.net/20.500.12185/350.

3 https://hdl.handle.net/20.500.12185/432.

4 https://2021.sacair.org.za/shared-task/ and https://dh2021.digitalhumanities.org.za/shared-task/.
